# Evaluation of contact angle and mechanical properties of resin monomers filled with graphene oxide nanofibers

**DOI:** 10.1590/0103-6440202305299

**Published:** 2023-10-27

**Authors:** Marilia Mattar de Amoêdo Campos Velo, Tatiana Rita de Lima Nascimento, Alyssa Teixeira Obeid, Nair Cristina Margarido Brondino, Rafael Francisco Lia Mondelli

**Affiliations:** 1 Department of Chemistry, Research and Extension Center for Fuels and Materials Laboratory (NPELACOM), Federal University of Paraiba, João Pessoa, Paraíba, Brazil; 2 Technical School of Health, Federal University of Paraíba, João Pessoa, Paraíba, Brazil; 3 São Paulo State University-UNESP, School of Science, Department of Mathematics, Bauru, São Paulo, Brazil.

**Keywords:** biomaterials, hybrids, nanofibers, graphene oxide, resin composite

## Abstract

This *in vitro* study synthesized hybrid nanofibers embedded in graphene oxide (GO) and incorporated them into experimental resin composite monomers to evaluate their physical-mechanical properties. Inorganic-organic hybrid nanofibers were produced with precursor solutions of 1% wt. GO-filled Poly (d,l-lactide, PLA) fibers and scanning electron microscopy (SEM) and energy-dispersive X-ray spectroscopy (EDS) characterized the morphology and chemical composition of the spun fibers. Resin composite monomers were developed and a total of 5% nanofibers were incorporated into the experimental materials. Three groups were developed: G1 (control resin monomers), G2 (resin monomers/PLA nanofibers), and G3 (resin monomers/inorganic-organic hybrid nanofibers). Contact angle (n=3), flexural strength (n=22), elastic modulus (n=22), and Knoop hardness (n=6) were evaluated. The mean of the three indentations was obtained for each sample. The normality of data was assessed by QQ Plot with simulated envelopes and analyzed by Welch's method (p<0.05). Overall, SEM images showed the regular shape of nanofibers but were non-aligned. Compositional analysis from EDS (n=6) revealed the presence of carbon and oxygen (present in GO composition) and Si from the functionalization process. The results of contact angle (°) and hardness (Kg/mm^2^) for each group were as follow, respectively: G1 (59.65±2.90; 37.48±1.86^a^), G2 (67.99±3.93; 50.56±1.03^b^) and G3 (62.52±7.40; 67.83±1.01^c^). The group G3 showed the highest Knoop hardness values (67.83 kg/mm^2^), and the flexural strength of all groups was adversely affected. The experimental resin composite composed of hybrid nanofibers with GO presented increased hardness values and hydrophilic behavior.

## Resumo

Este estudo *in vitro* sintetizou nanofibras híbridas embebidas em óxido de grafeno (GO), incorporando-as à uma resina composta experimental de monômeros para avaliar suas propriedades físico-mecânicas. Nanofibras híbridas inorgânica-orgânicas foram produzidas com soluções precursoras de fibras poli (d, l-lactídeo, PLA) preenchidas com GO a 1% em peso e microscopia eletrônica de varredura (MEV) e espectroscopia de raio-X de energia dispersiva (EDS) caracterizaram a morfologia e composição química das fibras. Monômeros de resina composta foram desenvolvidos e um total de 5% de nanofibras foi incorporado aos materiais experimentais. Três grupos foram desenvolvidos: G1 (monômeros de resina controle), G2 (monômeros de resina/ nanofibras de PLA) e G3 (monômeros de resina/nanofibras híbridas inorgânico-orgânicas). Ângulo de contato (n=3), resistência à flexão (n=22), módulo de elasticidade (n=22) e dureza Knoop (n=6) foram avaliados. A média das três endentações foi obtida para cada amostra. A normalidade dos dados foi avaliada pelo QQ Plot com envelopes simulados e analisada pelo método de Welch (p<0,05). No geral, as imagens de MEV mostraram forma regular de nanofibras, mas não alinhadas. A análise composicional de EDS (n=6) revelou a presença de carbono e oxigênio (presentes na composição do GO) e Si resultante do processo de funcionalização. Os resultados do ângulo de contato (°) e dureza (Kg/mm2) para cada grupo foram os seguintes, respectivamente: G1 (59,65±2,90; 37,48±1,86a), G2 (67,99±3,93; 50,56±1,03b) e G3 (62,52±7,40; 67,83±1,01c). G3 apresentou os maiores valores de dureza Knoop (67,83 kg/mm2), e a resistência à flexão de todos os grupos foi prejudicada. A resina composta experimental composta por nanofibras híbridas com GO apresentou maiores valores de dureza e comportamento hidrofílico.

## Introduction

Resin composite is currently the material of choice for restorations in anterior and posterior teeth, mainly due to its esthetic and adhesive properties ^1^ that allow for a minimal intervention procedure. Nonetheless, studies show a high annual failure rate of resin composite restorations and low clinical longevity compared to dental amalgam ^1^. Although caries risk and patient-level factors play a major role that affecting resin composite restoration longevity, such clinical failure can be also attributed to material properties and the stress generated by fatigue over time ^2^ ^,^ ^3^. Therefore, the high incidence of resin composite restoration replacements highlights the need to develop new restorative materials, with better physical-mechanical properties and also bioactive potential to prevent caries adjacent to the restoration ^3^ ^,^ ^4^.

Composite materials consist of a polymeric matrix in which inorganic particles are dispersed to improve physical and mechanical properties ^5^. However, inorganic fillers are more rigid than the resin matrix, so the stress during mastication is transmitted through these particles, producing small fractures and consequently weakening the resin matrix ^6^ ^,^ ^7^. In addition, resin composites that contain anticaries agents overall present insufficient mechanical properties, which may develop initiation sites for the propagation of flaws and cracks ^8^. 

Nanofibers have been used as a high-strength reinforcing phase of resin-based composites. Unlike conventional fillers, nanofibers are distributed and aligned uniformly ^7^ ^,^ ^9^, enhancing the intermolecular hydrogen bonding between the nanofibrous filler and the resin matrix ^10^. Several studies demonstrated that the reinforcement of the resin matrix with nanofibers composed of organic polymers improves the mechanical properties of resin-based composites ^7^ ^,^ ^11^ ^,^ ^12^ ^,^ ^13^. Recently, it was shown that the incorporation of hybrid nanofibers (composed of inorganic and organic phases) in a self-adhesive resin cement, adhesive system, varnish, and enamel resin infiltrate significantly enhanced the mechanical properties and impart bioactivity ^7^ ^,^ ^14^ ^,^ ^15^, since they also act as ion-releasing filler, being one of the most effective methods to reinforce anticaries agents. 

In 2022, Velo et al. ^16^, synthesized a resin composite matrix consisting of a clay mineral and graphene oxide (GO) nanoparticles. The physical-mechanical properties of the resin composite improved and calcium (Ca) and phosphorus (P) ions were deposited on the material surface, presenting a bioactive potential. GO is among the hardest materials available, with high mechanical strength, and high surface area, and its length may extend by up to 25%, a highly desirable property for developing dental materials ^17^ ^,^ ^18^. Owing to GO also induces nucleation and crystallization for hydroxyapatite growth, the development of a highly resistant resin composite which also presents bioactive potential could increase the marginal sealing with restorations and the long-term performance. Therefore, a resin composite filled with GO-hybrid nanofiber could overcome the issues related to the physical-mechanical properties of the material and such a composite would be suitable for further research in a biomimetic remineralization approach. 

Thus, the present *in vitro* study synthesized inorganic-organic hybrid nanofibers filled with GO and, incorporated them into experimental resin composite monomers to evaluate their physical and mechanical properties.

## Materials and methods

### Hybrid nanofiber fabrication

The hybrid nanofibers were fabricated with a modified air-blowing spinning technique, as described previously ^19^. A professional fixed airbrush cup (Model BC 61 - 7cc Reservoir) was used to produce fibers layer-by-layer with a 0.3-mm standard nozzle, obtaining a non-woven fiber mat system ^14^. The nanofibers consisted of Poly (D, L lactide) (PLA; Merck, Darmstadt, Germany) in pellet form, tetraethyl orthosilicate (TEOS precursor solutions; ≥ 99% purity, Merck, Darmstadt, Germany), and deionized water in a standard HCL solution at a 1:3:3 (mol/L) ratio. TEOS was used as a precursor for the synthesis of silica to functionalize the inorganic particles during the process with the PLA, turning it into organofunctional ^7^. Separately, a second polymer solution was prepared (20% in mass) with 10 wt.% of GO (relative to the total polymer mass), and sonification was performed for three hours at eight-minute intervals per hour. Next, the PLA mass was added to the initial solution, dripped onto the second polymer, and constantly stirred at room temperature for three hours to produce the inorganic-organic hybrid solution.

### Fiber characterization and sample preparation

A scanning electron microscope (SEM) coupled to energy-dispersive X-ray spectroscopy (EDS) assessed fiber morphology. The experimental resin monomers consisted of 49.5% BisGMA, 49.5% TEGDMA, 0.8% 2-dimethacrylate, and 0.2% camphorquinone. A rate of 5% of nanofibers composed of 1% GO was added slowly by manual mixing and homogenized for one minute ^16^, developing three groups: G1 (control resin monomers), G2 (resin monomers/PLA nanofibers), and G3 (resin monomers/PLA nanofibers/1% GO). All samples were light-activated with a third-generation LED device (VALO; Ultradent, South Jordan, Utah, USA) operating at 1,000 mW/cm^2^ for 40 seconds at a 1-mm distance. Wettability assessment with contact angle, three-point flexural strength, elastic modulus, and surface microhardness characterized the materials.

### Contact angle determination

Wettability was performed to evaluate material hydrophobicity after nanofiber functionalization. The samples (n=3) were separately mounted on glass microscope slides with a thin strip of Scotch-Magic™ Tape (3M). Droplets of distilled water (MILIQ) (3 µl) were inserted on each sample surface, and contact angles were obtained by averaging the results of three measurements per sample without repetition in the same sample area. Then, wettability capacity was recovered using KSV Instruments Ltd. equipment, model CAM101 ^16^.

### Flexural strength and elastic modulus analysis

Bar-shaped samples measuring 8×2×2 mm^2^ (n=6) were prepared in stainless-steel split molds according to ISO 4049, changing the sample length to prevent overexposure or uncured regions considering the diameter of the LED curing device ^7^ ^,^ ^16^. A universal testing machine (Instron 5943, Norwood, MA, USA) (500 N load-cell at 0.5 mm/min) was used, and the samples were loaded in a three-point bending apparatus with a 6-mm span length on the top surface of each specimen. Flexural strength and elastic modulus were calculated according to previous studies ^7^ ^,^ ^16^.

### Knoop hardness

Disc-shaped samples (10×2 mm^2^) (n=6) were prepared by inserting the material into stainless-steel molds and covered with polyester tape. The sample surface was polished with decreasing abrasive papers (#600-grit to #1200-grit, Buehler Ltd., Lake Bluff, IL, USA) for two minutes each, followed by a felt disc with a diamond suspension (Buehler Ltd., Lake Bluff, IL, USA) to obtain a flat and smooth surface suitable for evaluating hardness. Three indentations were made on the top surface of each specimen along a middle line spaced at 100 μm (Knoop diamond, 50 g, dwell-time 10 s) with digital microhardness equipment (Micromet II, Buehler, USA). The mean of the three readings was recorded for each sample ^7^ ^,^ ^16^.

### Statistical analysis

The data were statistically analyzed with R software (R Core Team, 2020). Normality was assessed with a QQ Plot with simulated envelopes. Paired comparison tests were performed between groups with sandwich variance estimators. For contact angle analysis, Welch’s ANOVA test was performed (p<0.05). The significance level was preset at 𝛼=0.05. Qualitative analyses were only described.

## Results

**Figure 1:**
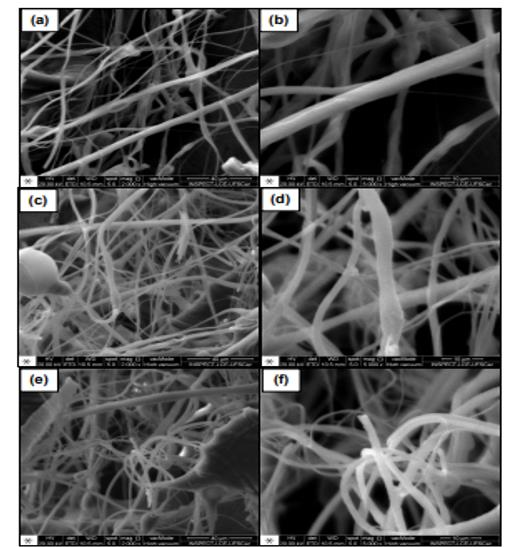
SEM images of PLA fibers morphology: (a), (c), (e) under 2000x magnification and, (b), (d), (f) under 5000x magnification.

**Figure 2:**
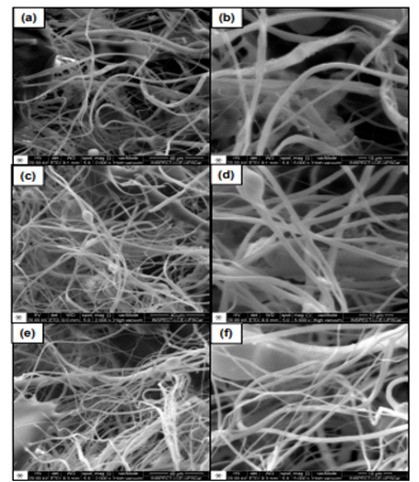
SEM of PLA/1% graphene oxide fibers morphology: (a), (c), (e) under 2000x magnification and, (b), (d), (f) under 5000x magnification.

**Figure 3:**
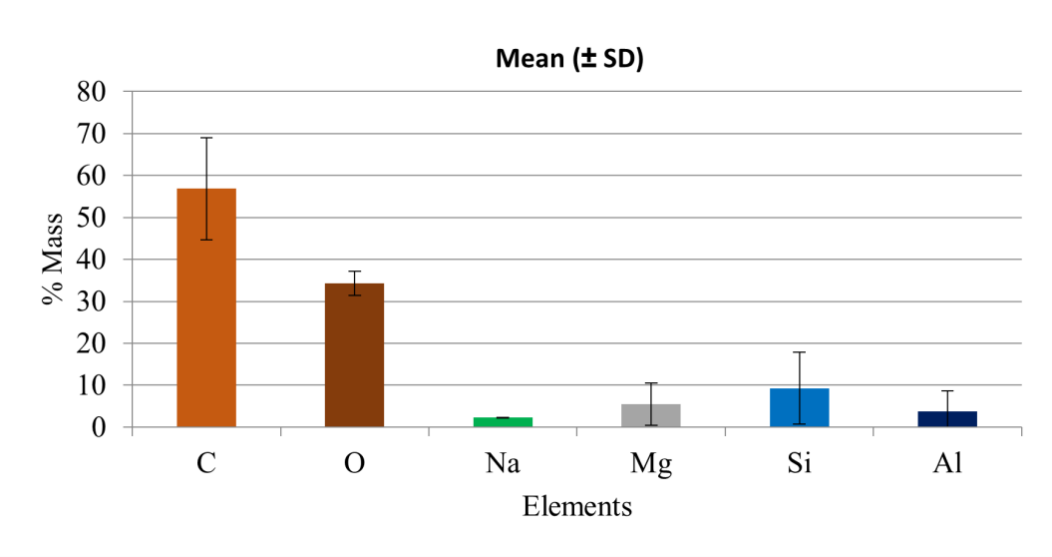
Element content in mass (%) on the PLA/1% graphene oxide fibers developed.

SEM images assessed the morphology of GO nanofibers (Figures 1 and 2), showing non-alignment, irregular shape, or preferred alignment. There were also some agglomerations. There was Si (Figure 3) in the nanofiber compositions, demonstrating the functionalization process of the compound. Overall, hybrid nanofibers filled with GO consisted of carbon (C) and oxygen (O), elements that compose GO, as shown in Figure 3.

Table 1 shows the contact angle results. According to the literature, angles smaller than 90° are hydrophilic materials, and higher than 90° are hydrophobic ^20^. The developed resin composite filled with GO hybrid nanofibers showed hydrophilic behavior, although there was no statistical difference among the groups (p=0.13). 

**Table 1:** Values (mean±SD) of the contact angle (°), microhardness (Kg/mm^2^), flexural strength (MPa), and elastic modulus (GPa).

Groups	Contact angle (n=3)	Microhardness (n=6)	Flexural strength (n=22)	Modulus of elasticity (n=22)
G1	59.65±2.90^a^	37.48±1.86^a^	79.27±18.91^b^	3.22±0.29^b^
G2	67.99±3.93^a^	50.56±1.03^b^	43.16±9.00^a^	2.80±0.24^a^
G3	62.52±7.40^a^	67.83±1.01^c^	54.48±10.18^b^	3.25±0.22^b^

Values in the same column with different superscript lower-case letters significantly differ from each other (p<0.05).

When evaluating microhardness, the values increased with nanofiber incorporation (G2 and G3). The group composed of GO hybrid nanofibers presented the highest hardness values (p < 0.0001). As for flexural strength, the values decreased with PLA nanofiber incorporation, and there was no difference between the control group (G1) and the group with GO hybrid nanofibers (G3) (p=0.88). The elastic modulus of G3 also did not differ from the control group (G1) (p=0.65).

## Discussion

Improvements in the physical and mechanical properties of restorative materials have been highlighted to ensure the long-term clinical success of restorations ^21^. Using inorganic-organic hybrid nanofibers can be a promising strategy to improve the mechanical properties of several dental materials ^7^ ^,^ ^15^ and such hybrid nanofibers are suitable scaffolds to contain bioactive inorganic fillers that can release ions, such as GO.

In the current study, nanofibers composed of 1% GO were developed based on previous studies. It was demonstrated that adding 0.1-5% ^22^ by weight of graphene as a nanofiller in polymer-based composites would be sufficient to consistently improve the mechanical properties of resin-based composites. The surface functionalization of hybrid nanofibers was carried out to turn it organofunctional and enhance the adhesion with the organic matrix of the monomers ^7^. Overall, the functionalization process maintains the nanostructure chemically stable avoiding agglomeration, and, consequently, improves their mechanical properties ^7^ ^,^ ^23^ ^,^ ^24^.

Despite the functionalization process of the nanofibers that occurred as demonstrated in the EDS by the presence of Si (Table 1), some clusters were developed (Figure 2). Although a small amount of GO can improve the polymer’s properties as demonstrated in other studies, the major challenge is to avoid agglomeration of GO into the matrix, which could be prevented by the surface modification of graphene nanoparticles ^25^. However, in the current research, non-functionalized GO nanoparticles were used to prepare the nanofibers which might have influenced the results due to the agglomeration within the nanofibers, decreasing flexural strength values (Figure 2, Table 1). Overall, GO sheets present strong hydrophobicity, interfering in the dispersion process within the matrix, which aggregates GO ^26^. We hypothesized that if functionalized GO nanoparticles were used to prepare the nanofibers, clusters were not formed, improving the flexural strength of the resin monomers, but other studies should be conducted to conclude it. 

Our results are in contrast with some previous studies, which demonstrated that incorporating nanofibers into monomers usually increases flexural strength ^6^ ^,^ ^7^ ^,^ ^12^ ^,^ ^27^ ^,^ ^28^. As discussed above, the functionalization process of fillers highly interferes with the improvement of mechanical properties and polymerization percentage of resin-based materials, owing to the organic matrix-inorganic filler interface bond formed by silane structures ^29^. Moreover, resistance to crack propagation may be a concern fiber and matrix-related properties when fibers are longer than their critical length ^20^. In this case, resin composites are more effective in transferring stress from the matrix. These specific features may interfere with the flexural properties of the composite material and enhance its resistance to fracture ^30^.

Conversely, microhardness increased for G2 and G3, which can be attributed to the resistance of nanofibers and GO structures, respectively. Overall, hardness is relevant to predicting the wear behavior and durability of the material and, can be influenced by the type of fillers, shape, and distribution within the matrix ^31^. Nanofibers present an extremely small diameter and large surface area, which significantly increases strength ^32^, thus explaining higher hardness values for G2 composed of PLA nanofibers. The presence of GO hybrid nanofibers in G3 justifies the highest hardness values (Table 1) due to their excellent physical and mechanical properties. Several factors can affect the hardness of a resin-based material, including filler size, shape fraction in the inorganic phase, and the specific composition and structure of the organic matrix ^33^. Hardness usually increases with filler content ^34^. While the volume fraction of the filler increases, a point is reached where particles are in mutual contact within the matrix, transferring this stress point across the material predominantly via (hard) particle-particle interactions ^30^.

The contact angle results showed the hydrophilic behavior of the studied monomers, mainly attributed to silica, which improves GO dispersion and reduces material hydrophobicity, a significant property to facilitates ion exchange and the bioactive potential of the biopolymer ^35^. There was no difference among all tested groups about contact angle analysis (p>0.05, Table 1). However, as a limitation of the present study, the small sample size evaluated (n=3) tends not to reject the null hypothesis of equality of means between groups, even when this difference exists; thus, such results should be analyzed with caution. Even though, it should be mentioned that contact angle values less than 90° characterize hydrophilic materials, the more soluble the material is, the more bioactive potential it presents. 

The contact angle of a surface is also related to bacterial adhesion and, clinically, bacterial adhesion occurs mainly on hydrophobic surfaces ^12^ ^,^ ^36^. Therefore, further investigations should be conducted with GO fillers functionalized to prepare the nanofibers and test the bioactive potential and antimicrobial effect of this material. A resin composite presenting improved physical-mechanical properties and bioactive and/or antimicrobial properties shows potential for future applications as a restorative material.

In conclusion, the experimental resin composite with GO hybrid nanofibers presented increased hardness and hydrophilic behavior, representing a promising strategy for ion exchange in biomimetic conditions. 
